# Pre-Ramadan time-below-range stratifies the simulated pump basal-rate dose–response during Ramadan fasting: a five-policy paired virtual-patient study

**DOI:** 10.3389/fendo.2026.1922149

**Published:** 2026-09-04

**Authors:** Ameen Banjar

**Affiliations:** Department of Information Systems and Technology, College of Computer Science and Engineering, University of Jeddah, Jeddah, Saudi Arabia

**Keywords:** basal insulin, continuous glucose monitoring, dose response, precision dosing, ramadan, treatment heterogeneity, type 1 diabetes, virtual patients

## Abstract

**Background:**

Pump basal-rate reduction during Ramadan may prevent hypoglycemia but can increase hyperglycemia. Its dose-response relation and phenotype-dependent trade-off remain uncertain.

**Methods:**

Thirty UVA/Padova virtual patients (10 children, 10 adolescents, 10 adults) underwent paired 30-day Ramadan conditions delivering 100%, 90%, 80%, 70%, or 60% of the pump steady-state basal rate. The primary complete-case analysis included 29 virtual patients. Outcomes were time in range (TIR), time below range (TBR), time above range (TAR, including TAR *>* 250 and TAR *>* 300), and rescue corrections. Paired permutation tests and bootstrap intervals quantified effects. Robustness and revision analyses used three sensor seeds, three seeded meal/timing scenarios with a held-out evaluation, a reactive-correction sensitivity analysis, a time-of-day decomposition, terminal-event reporting, and a negative-control (sham) test of the pre-Ramadan marker.

**Results:**

Mean TIR was 81.7%, 79.9%, 73.9%, 65.3%, and 56.0% at 100%, 90%, 80%, 70%, and 60%, respectively (*P <*0.001). Compared with 100%, 90% basal reduced TBR by 1.6 percentage points (95% CI −2.1 to −1.2) while changing TIR by −1.8 points (95% CI −3.7 to −0.3; Holm-adjusted *P* = 0.031). Severe hyperglycemia rose steeply with deeper reduction (TAR*>*250: 2.9% at 100% to 14.8% at 60%). Enabling realistic reactive corrections raised TIR at the deepest policies but preserved the same policy ordering. Pre-Ramadan TBR stratified the TIR response across reduced policies (omnibus patient-blocked permutation *P* = 0.023), with the strongest gradients at 70% and 60% basal; a negative-control analysis showed this signal was not specific to TBR but reflected a broader baseline-glycemia pattern. In a held-out scenario, a revised safety-first selection rule improved hypoglycemia-target attainment (39% to 79%) and reduced TBR by 2.9 points but incurred a 5.2-point TIR cost, demonstrating that prioritizing fasting safety involves a glycemic trade-off.

**Conclusion:**

Ramadan pump basal-rate reduction should be treated not as a uniform percentage change but as a phenotype- and context-dependent trade-off. Among the reduced policies, 90% basal produced the smallest TIR loss while lowering hypoglycemia; pre-Ramadan TBR was an exploratory candidate stratifier; and meal-size associations differed by policy. These mechanistic findings apply to simulated pump basal-rate modulation and require prospective, CGM-anchored validation before any clinical use.

## 1 Introduction

Ramadan is observed by more than a billion Muslims worldwide, and a large fraction of people with diabetes choose to fast despite religious exemptions on medical grounds. In the landmark population-based EPIDIAR survey of 13 countries, 42.8% of people with type 1 diabetes fasted for at least 15 days during Ramadan, yet fewer than half of insulin-treated patients adjusted their dose (1); many therefore fast against medical advice and with unmodified regimens. The fast reorganizes the entire daily rhythm of eating, insulin exposure, sleep, and physical activity: people abstain from food and fluids from dawn (suhoor) until sunset (iftar), after which intake is concentrated into a few evening and predawn hours. For a person with type 1 diabetes, this creates two opposing hazards within a single day. The prolonged daytime fast, sustained by exogenous basal insulin in the absence of carbohydrate, predisposes to hypoglycemia, whereas the abrupt, carbohydrate-dense iftar predisposes to marked postprandial hyperglycemia (2).

International guidance, most prominently the IDF–DAR practical guidelines, therefore emphasizes pre-Ramadan risk stratification, structured education, continuous glucose monitoring (CGM), individualized insulin adjustment, and explicit criteria for breaking the fast (2). Structured risk-stratification tools built on this guidance have since been validated in primary-care populations (3). Real-world CGM studies during Ramadan have repeatedly documented the resulting glycemic disturbance: broader diabetes cohorts show substantial hyperglycemia (4), people with type 1 diabetes show improved Glycemia Risk Index scores only under structured fasting plans (5), glycemic deterioration is greatest among those with suboptimal baseline control (6), and hybrid closed-loop therapy benefits higher-risk fasters (7). Notably, observational CGM cohorts have shown that, even with advanced monitoring, the largest glucose excursions occur after iftar and suhoor, indicating that peri-meal insulin adjustments remain insufficient in routine practice. Automated insulin delivery has emerged as a particularly promising approach: case reports, randomized trials, and pilot cohorts of children, adolescents, and adults using hybrid closed-loop systems during Ramadan have shown that automated basal modulation can maintain time in range while limiting hypoglycemia (8–12).

Within this multifactorial picture, reduction of the basal insulin rate is the single most widely recommended adjustment for mitigating daytime fasting hypoglycemia, and reductions of roughly 20% to 40% are frequently suggested as defaults. Yet no single reduction is appropriate for every person, and the isolated effect of basal reduction is difficult to quantify clinically because it never occurs in isolation: it is bundled with carbohydrate counting, post-iftar bolus strategy, correction logic, education, and device choice (13–17). A randomized pilot study, for example, showed that alternate pump settings and advanced bolus features improved time in range (TIR) more than basal adjustment alone (13), while structured education with advanced monitoring allowed selected people with type 1 diabetes to fast safely (14). Disentangling the dose-response contribution of basal reduction itself, and identifying who tolerates it, is consequently an open question that randomized designs cannot easily resolve, because the same individual cannot be assigned several basal policies under identical conditions.

In silico experiments with validated type 1 diabetes simulators offer a controlled route to this question. The UVA/Padova simulator, accepted by the US Food and Drug Administration (FDA) in lieu of animal testing for closed-loop algorithm development, allows the same virtual physiology to undergo alternative insulin policies with identical meals, timing, and sensor noise (18–21). Such experiments are mechanistic and must not be equated with clinical evidence or with validated patient-specific digital twins (22, 23); they cannot establish efficacy. They can, however, isolate the dose-response contribution of a single policy lever, quantify treatment-effect heterogeneity across a virtual cohort, and generate falsifiable hypotheses for prospective testing—mirroring the broader shift toward precision medicine that frames patient heterogeneity as a target for individualized treatment rather than a nuisance to be averaged away (24).

### Current gaps

Three gaps motivate this work. First, prior in silico and clinical Ramadan studies have typically compared a single reduced basal policy (commonly a 30% reduction) against no reduction, leaving the full dose-response relation across the clinically used range uncharacterized; the shape of the hypoglycemia–hyperglycemia trade-off, and the location of any favorable region, are therefore unknown. Second, although guidelines call for individualized adjustment, no quantitative, CGM-derived phenotype has been shown to identify which patients tolerate basal reduction with the least loss of TIR. Third, the interaction between basal reduction and the size of the iftar carbohydrate load—whether meal moderation matters more under some policies than others—has not been formally tested. Addressing these gaps requires applying many basal policies to the same physiology under matched conditions, which is feasible only in silico.

### Study hypotheses

We extended an initial two-policy comparison (100% versus 70% basal), which had suggested that pre-Ramadan hypoglycemia burden was more informative than age group for identifying a favorable trade-off, to a systematic five-policy design delivering 100%, 90%, 80%, 70%, or 60% of basal. We prespecified three hypotheses: (i) larger basal reductions would progressively decrease time below range (TBR) but worsen TIR, time above range (TAR), and rescue-treatment burden, producing a graded trade-off rather than a single optimum; (ii) elevated pre-Ramadan TBR would modify the TIR response, identifying virtual patients with a less adverse reaction to reduction; and (iii) the association between carbohydrate load and TIR would depend on the basal policy, being greatest where basal delivery remained near-adequate.

## 2 Materials and methods

### 2.1 Study design and virtual cohort

This paired in silico crossover study used all 30 UVA/Padova virtual patients distributed with the open-source *simglucose* implementation of the FDA-accepted simulator (25): 10 children, 10 adolescents, and 10 adults. The UVA/Padova model represents glucose-insulin dynamics through a system of ordinary differential equations describing glucose subsystems, insulin absorption and action, endogenous glucose production, renal excretion, and gut absorption, with patient-specific parameters distributed across the cohort to span a realistic range of body weight, insulin sensitivity, insulin-to-carbohydrate ratio (ICR), and correction factor. Because every virtual patient is a fixed parameter set, the same physiology can be subjected to multiple insulin policies under otherwise identical conditions, which is the property that makes a paired dose-response design possible. Throughout, the manipulated variable is the continuous *pump* basal rate; the design therefore models basal-rate modulation as delivered by insulin-pump therapy and does not represent dose reduction of long-acting basal insulins (e.g., glargine, degludec) used in multiple daily injection (MDI) regimens, whose pharmacodynamics and adjustability differ fundamentally.

Each virtual patient underwent a 30-day pre-Ramadan reference condition and five 30-day Ramadan policies delivering 100%, 90%, 80%, 70%, or 60% of the model-specific basal rate. The pre-Ramadan reference is a full 30-day run immediately preceding the Ramadan window that uses the same virtual physiology, the same 100% basal rate, and the same insulin-delivery logic (including the below-70 mg/dL suspension and disabled routine corrections) as the Ramadan arms, but with a conventional non-fasting meal pattern (three main meals and a snack distributed across the daytime) rather than the suhoor–iftar concentration. Pre-Ramadan TBR therefore arises purely from each patient’s own basal requirement interacting with this conventional schedule under an identical CGM sensor-noise seed, and is available before the fast in the same way a clinician would read a patient’s pre-Ramadan CGM. The 100% Ramadan condition shares the patient’s physiology and meal protocol but differs from the pre-Ramadan reference in meal timing and distribution, isolating the effect of the Ramadan schedule itself; the four reduced policies then isolate the effect of basal reduction within the Ramadan schedule. A Dexcom CGM sensor model (Dexcom, Inc.) and the generic InsulinPump simulation model implemented in simglucose (not representing a specific commercial pump manufacturer) were used at 3-minute sampling intervals. Basal insulin was calculated from each model’s steady-state basal rate (u2ss) and body weight; simulator-supplied carbohydrate ratios and correction factors were retained unchanged so that the only manipulated variable was the basal multiplier. The same CGM sensor-noise seed was used within each virtual patient across paired policies, so that between-policy differences could not arise from differing sensor-noise realizations. Cohort characteristics by age group are summarized in **Supplementary Table 1**.

### 2.2 Meal and insulin scenarios

Meal scheduling was designed to reflect the documented structure of Ramadan intake, in which carbohydrate is concentrated into the predawn suhoor and the postsunset iftar with an evening snack between. Daily carbohydrate targets were 150 g for children, 220 g for adolescents, and 250 g for adults, consistent with age-appropriate intake. Ramadan carbohydrate was allocated to suhoor (35%), a two-stage iftar (an initial light fastbreaking portion of 10% followed by a main meal of 40%), and an evening snack (15%). To represent realistic day-to-day variability in intake, carbohydrate exposure followed a fixed 10-level sequence ranging from 70% to 130% of the age-group target, and suhoor timing varied among three prespecified offsets relative to dawn. The sequence was identical across paired policies, ensuring that any between-policy difference reflected the policy rather than the meal stream.

Meal insulin was calculated from the announced carbohydrate and the patient-specific ICR, then distributed across the modeled eating interval. Routine reactive correction boluses were disabled so that the basal contrast was not confounded by a reactive controller; this is a deliberate simplification that isolates the basal lever at the cost of clinical realism, and its impact is quantified separately in a realistic-correction sensitivity analysis (see Statistical analysis). Basal and bolus delivery were suspended below 70 mg/dL to emulate a predictive-low-glucose safety response. A conservative rescue correction was permitted at CGM ≥ 300 mg/dL, targeting 250 mg/dL, and separated by at least 4 h, emulating a cautious fast-preserving correction rather than aggressive normalization. In the primary analysis, this rescue dose was capped at a uniform 1 U purely as an isolation safeguard—to prevent a large reactive bolus from masking the basal contrast—rather than as a clinically calibrated dose; because a fixed 1 U cap is not equivalent across body sizes, the sensitivity analysis instead applies a patient-specific dose ceiling (below).

### 2.3 Outcomes and estimands

The primary outcome was TIR (70–180 mg/dL), consistent with international CGM consensus recommendations (26). Secondary outcomes were TBR below 70 mg/dL, TBR below 54 mg/dL, TAR above 180 mg/dL, mean CGM, glucose management indicator (GMI), coefficient of variation (CV), low and high blood glucose indices, and rescue-correction burden. These outcomes overlap substantially with the components of the Glycemia Risk Index, a composite hypoglycemia/hyperglycemia metric increasingly used to summarize CGM-based glycemic risk (27).

The primary estimand was the mean within-patient difference between each reduced policy and Ramadan 100% over 30 days among virtual patients completing all five policies. A *terminal run* was defined by the simulator’s physiological stopping condition, namely, blood glucose exceeding 600 mg/dL or falling below 10 mg/dL, at which point the run was halted; such runs were retained as separate safety outcomes rather than as truncated continuous outcomes. A sensitivity analysis included all 30 virtual patients over the first nine fully observed days, the longest complete horizon available under every policy.

### 2.4 Statistical analysis

Daily outcomes were averaged within virtual patient and policy. Overall policy effects were tested with the Friedman test. Each reduced policy was then compared with 100% using two-sided paired sign-flip permutation tests of the mean difference (200,000 random permutations), with Holm adjustment across the four comparisons within each outcome. Mean paired differences received 20,000-replicate patient-level percentile-bootstrap 95% confidence intervals. Because the confidence intervals are unadjusted percentile-bootstrap intervals whereas the *P* values arise from multiplicity-adjusted paired permutation tests, an interval excluding zero may occasionally accompany an adjusted *P* above 0.05; the two quantities answer different inferential questions and are reported together for transparency.

The phenotype analysis included exploratory policy-specific correlations and an exploratory formal test of effect modification. Exploratory Spearman correlations related pre-Ramadan TBR to the TIR effect at each reduced policy, and pre-Ramadan TBR was dichotomized at 4%, the consensus upper target for TBR below 70 mg/dL (26). To test effect modification formally, the within-patient TIR change from the 100% policy, ΔTIR_*ip*_ = TIR_*ip*_ − TIR_*i*_,_100_, was regressed on centered pre-Ramadan TBR separately within each reduced policy *p* ∈ {90, 80, 70, 60}, and the omnibus null hypothesis that all four per-policy slopes equal zero was tested by a patient-blocked permutation test: the pre-Ramadan TBR vector was permuted *across* the 29 patients while each patient’s four policy rows were kept together (20,000 permutations), with the sum of squared per-policy slopes as the statistic. Per-policy slopes received 20,000-replicate patient-level bootstrap 95% confidence intervals. Finally, a *post hoc* hypoglycemia-target-constrained policy-selection analysis identified the policy with the highest TIR among those meeting both TBR ≤4% and TBR below 54 mg/dL ≤1%. If no policy met both targets, the highest-TIR policy was selected. This rule is included only to illustrate how phenotype-stratified simulation outputs might be summarized; it is descriptive, was not validated, and is not proposed as a deployable dosing algorithm.

As a sensor-noise robustness check, each complete-case patient was re-simulated under all five policies using two additional independent CGM sensor-noise seeds (three seeds per patient-policy pair), preserving the paired meal and timing schedule; outcomes were seed-averaged and the dose-response and phenotype analyses repeated. A descriptive within-patient (across-seed) variance ratio was computed per outcome and policy, treated as descriptive rather than a formal variance component.

Five additional analyses, conducted during revision in response to peer review, further probed robustness and inference. First, a *realistic-correction sensitivity analysis* re-ran all five policies with reactive corrections enabled: a correction was given when CGM ≥250 mg/dL, dosed toward a conservative 180 mg/dL target using each patient’s correction factor, separated by a 4-hour refractory interval, and capped by a patient-specific ceiling scaled to basal requirement rather than a fixed 1 U. Second, a *time-of-day decomposition* split each Ramadan day into fasting-daytime (05:00–18:00), post-iftar (18:00–22:00), nocturnal (22:00–01:00), and suhoor (01:00–05:00) windows and summarized TIR, TBR, TAR, and TAR*>*250 within each, together with the hourly distribution of hypoglycemia. Third, we report *policy-specific terminal-event rates*, the time to termination, and a worst-case sensitivity analysis in which the unobserved remainder of a terminated run was treated as lost horizon. Fourth, a *negative-control (sham) test* repeated the omnibus effect-modification test using non-TBR pre-Ramadan markers (TAR, HBGI, LBGI, TIR) to test whether the pre-Ramadan TBR signal is specific or reflects a generic floor effect. Fifth, a *lexicographic safety-first selection rule* first restricted to policies meeting hypoglycemia safety thresholds (TBR *<* 70 ≤4%, TBR *<* 54 ≤1%, no terminal event) and then maximized TIR among them, and was compared with the TIR-first rule above. Severe hyperglycemia was additionally summarized as TAR *>* 250 and TAR *>* 300.

### 2.5 Independent scenario and fasting-window extension

To test meal/timing uncertainty, the complete-case cohort was re-simulated under three independently seeded 30-day Ramadan scenarios, with the same meal realization and sensor seed paired across policies within each patient and scenario. Daily carbohydrate followed a log-normal multiplier (log-scale SD 0.18, truncated to 65–145%); suhoor, iftar, and snack times were jittered by up to 45, 20, and 45 min, and their carbohydrate shares drawn from a Dirichlet distribution centered on 35%, 50%, and 15%.

The five full-day policies were repeated, together with a fasting-window 80% policy that delivered 80% basal from the end of suhoor until the start of iftar and 100% otherwise. Outcomes were first averaged across the three scenarios within each patient, using only patient-scenario blocks that completed all six policies and reporting terminal runs separately. A scenario-held-out policy analysis trained a locked selection rule on scenarios 1–2 and evaluated it on scenario 3 against 100% basal. We evaluated two locked rules. The first, a TIR-first comparator, selected the highest-TIR full-day policy satisfying the consensus CGM targets (TBR ≤4%, TBR below 54 mg/dL ≤1%, TAR ≤25%) and, if none qualified, fell back to the highest-TIR policy. The second—our primary, safety-first rule, adopted in response to review—kept the same target-satisfying step but, when no policy qualified, ordered candidates lexicographically by lowest TBR below 54 mg/dL, then lowest TBR, then lowest TAR, and only then highest TIR, thereby never selecting a policy on TIR grounds alone. This tested scenario transportability within the same virtual patients, not external clinical validity.

### 2.6 Meal-size sensitivity analysis

To assess whether the basal dose-response was robust to meal size, we exploited the prespecified 10-level carbohydrate sequence (70–130% of the age-group target) embedded in the Ramadan protocol, using the same primary-engine output and the same 29 complete-case virtual patients as the main analysis. For each patient and policy, daily TIR was stratified by the day’s carbohydrate level (normalized to the patient’s age-group target), and the within-patient difference between low-carbohydrate days (≤85% of target) and high-carbohydrate days (≥110%) was computed and summarized with a 20,000-replicate patient-level percentile-bootstrap 95% confidence interval. A policy-dependent ordered trend in the within-patient low-minus-high carbohydrate TIR contrast was tested using a patient-blocked permutation test of Spearman’s𝜌 between basal policy and the contrast (20,000 permutations); policy labels were shuffled only *within* each virtual patient’s five observations, preserving the repeated-measures structure. This tests an ordered trend across policies rather than every form of categorical interaction. This analysis was prespecified through the embedded carbohydrate design rather than selected *post hoc*.

### 2.7 Ethics and reproducibility

Only virtual patients and generated data were used; institutional review board approval and informed consent were not applicable. The simulation and analysis code, compact derived datasets, and generated figures are publicly archived as software release v5.0.0 (28). Large raw simulation trajectories can be regenerated from the archived scripts and fixed seeds.

## 3 Results

### 3.1 Completeness and dose response

Twenty-nine virtual patients completed 30 days under all five policies and formed the primary cohort. One model, child#008, exhibited protocol-dependent terminal behavior under all reduced-basal policies: it completed the 100% policy (albeit with extreme physiological glucose, 15–591 mg/dL) but reached the simulator’s stopping threshold during day 26 at 90%, day 25 at 80%, day 25 at 70%, and day 10 at 60% (**Supplementary Table 2**). Because it did not complete all five 30-day policies, it was excluded from the paired complete-case analysis and is reported here as a separate safety signal rather than as truncated continuous outcomes.

**Figure 1:**
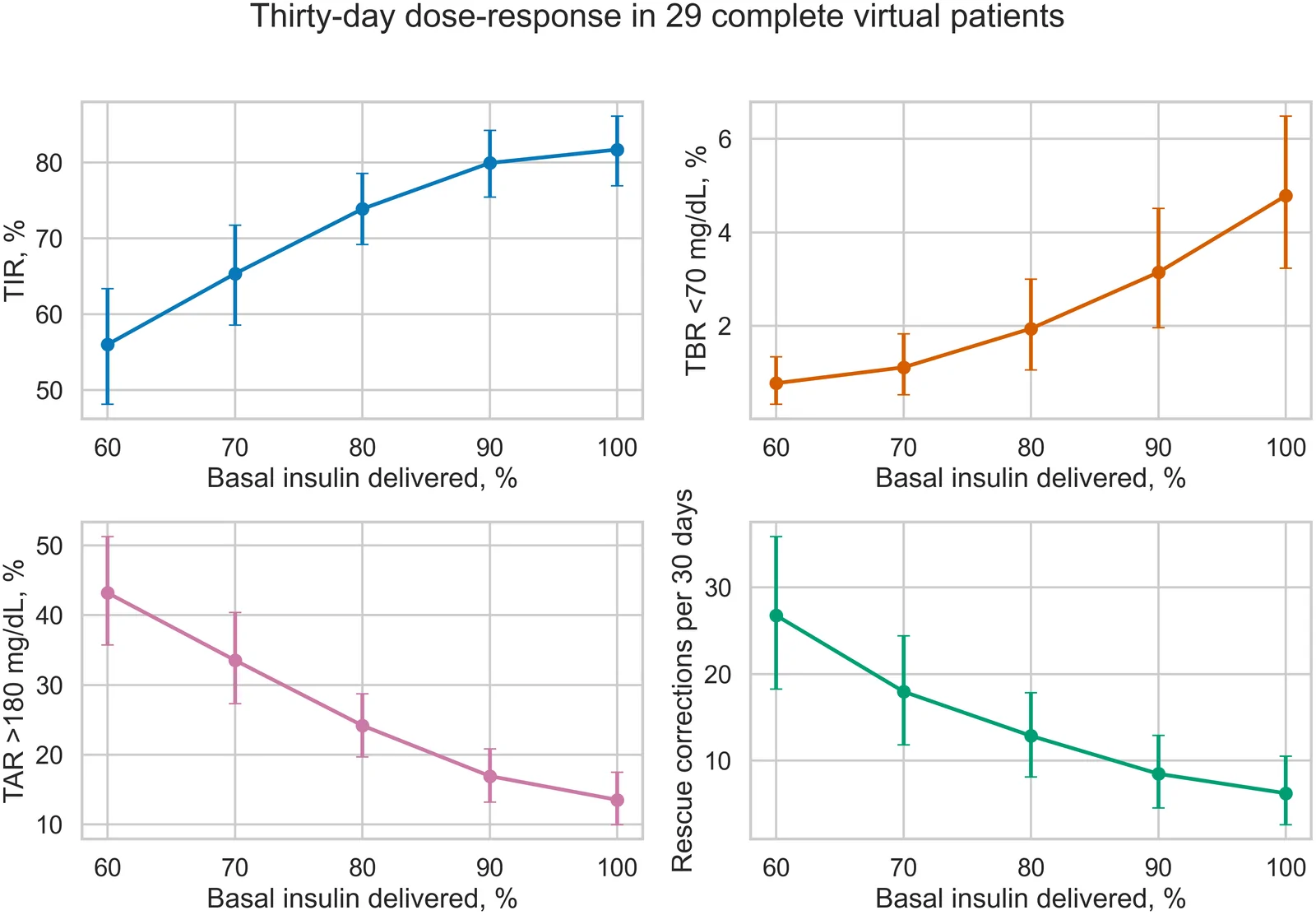
Dose-response of primary glycemic outcomes. Points are means and error bars are patient-level bootstrap 95% confidence intervals.

TIR, TBR, TAR, and rescue corrections all differed across policies (Friedman *P <*0.001 for each). Mean TIR declined monotonically across the five policies—81.7%, 79.9%, 73.9%, 65.3%, and 56.0% at 100%, 90%, 80%, 70%, and 60% basal, respectively—while TBR fell from 4.8% to 0.8% over the same range. The decline in TIR was markedly non-linear: the step from 100% to 90% cost only 1.8 percentage points, whereas each subsequent 10% reduction cost progressively more (6.0, 8.6, and 9.3 points), so that the benefit of avoiding hypoglycemia was bought at an accelerating price in TIR. TAR rose in mirror image from 13.5% to 43.2%, and rescue corrections more than quadrupled, from 6.2 to 26.8 per 30 days, indicating that the lower policies did not merely shift exposure but also imposed a rising treatment burden (**Table 1**; **Figure 1**). Mean CGM glucose and the glucose management indicator increased correspondingly, while the coefficient of variation changed little, indicating that the deterioration was driven by an upward shift in central glucose rather than by greater variability.

**Table 1:** Thirty-day outcomes across five basal insulin policies.

Outcome	100%	90%	80%	70%	60%
TIR, %	81.7 (12.9)	79.9 (12.5)	73.9 (13.1)	65.3 (18.2)	56.0 (21.7)
TBR *<* 70, %	4.8 (4.5)	3.1 (3.6)	1.9 (2.7)	1.1 (1.8)	0.8 (1.4)
TBR *<* 54, %	1.1 (1.8)	0.6 (1.2)	0.3 (0.7)	0.1 (0.3)	0.1 (0.2)
TAR *>* 180, %	13.5 (10.5)	16.9 (10.8)	24.2 (12.6)	33.5 (18.2)	43.2 (21.8)
Mean CGM, mg/dL	131.6 (14.9)	140.7 (14.6)	152.1 (17.4)	165.3 (23.7)	180.7 (32.2)
GMI, %	6.5 (0.4)	6.7 (0.4)	7.0 (0.4)	7.3 (0.6)	7.6 (0.8)
CV, %	31.4 (7.9)	30.8 (7.9)	30.6 (7.9)	30.1 (7.6)	29.7 (8.2)
Rescue corrections, n	6.2 (11.2)	8.5 (11.7)	12.9 (13.8)	18.0 (17.5)	26.8 (24.6)

Values are mean (SD) among 29 complete virtual patients.

### 3.2 Paired policy contrasts

The 90% policy reduced TBR by 1.6 points but changed TIR by only −1.8 points (Holm-adjusted permutation *P* = 0.031). At 80%, the mean TIR loss was 7.8 points and TAR increased by 10.7 points. Larger reductions produced progressively greater TIR loss, hyperglycemia, and rescue burden (**Table 2**).

**Table 2:** Paired differences from the Ramadan 100% policy.

Policy	TIR difference [95% CI]	TBR difference [95% CI]	TAR difference [95% CI]	Rescue difference [95% CI]
90%	−1.8 [−3.7, −0.3]	−1.6 [−2.1, −1.2]	3.4 [2.1, 5.2]	2.2 [0.8, 4.2]
80%	−7.8 [−12.6, −3.7]	−2.8 [−3.7, −2.0]	10.7 [6.7, 15.0]	6.6 [3.2, 10.5]
70%	−16.4 [−24.0, −9.4]	−3.7 [−4.9, −2.6]	20.0 [13.7, 27.3]	11.7 [6.5, 17.9]
60%	−25.7 [−34.8, −17.4]	−4.0 [−5.4, −2.7]	29.7 [21.9, 38.3]	20.5 [12.3, 30.2]

Differences are reduced policy minus 100%. Holm-adjusted permutation *P values* for TIR were 0.031, 0.002, *<* 0.001, and *<* 0.001, respectively. Adjusted *P <*0.01 for every displayed TBR, TAR, and rescue-correction contrast.

### 3.3 Pre-Ramadan TBR stratified the TIR response

Higher pre-Ramadan TBR was associated with a less adverse TIR response at 90% ( ρ=0.54, P=0.002), 80% ( ρ=0.54, P=0.002), 70% ( ρ=0.51, P=0.005), and 60% basal ( ρ=0.40, P=0.033). An exploratory formal test supported this pattern: the omnibus patient-blocked permutation test of the four per-policy pre-Ramadan TBR slopes provided evidence of effect modification within the simulator cohort ( P=0.023). The modification was concentrated at the larger reductions: the slope of Δ TIR on pre-Ramadan TBR (with unadjusted patient-level bootstrap 95% confidence intervals) was 0.13 points per 1% TBR at 90% ( −0.43 to 0.47), 0.91 at 80% ( −0.00 to 1.78), 1.74 at 70% (0.62 to 3.09), and 2.10 at 60% (0.80 to 3.69), the last two intervals excluding zero. Consistent with this, among 18 virtual patients with pre-Ramadan TBR below 4%, mean TIR differences were −2.1, −10.8, −22.4, and −33.0 points at 90%, 80%, 70%, and 60%, respectively, whereas among 11 with TBR at least 4% they were −1.2, −2.9, −6.5, and −13.7 points.

**Figure 2:**
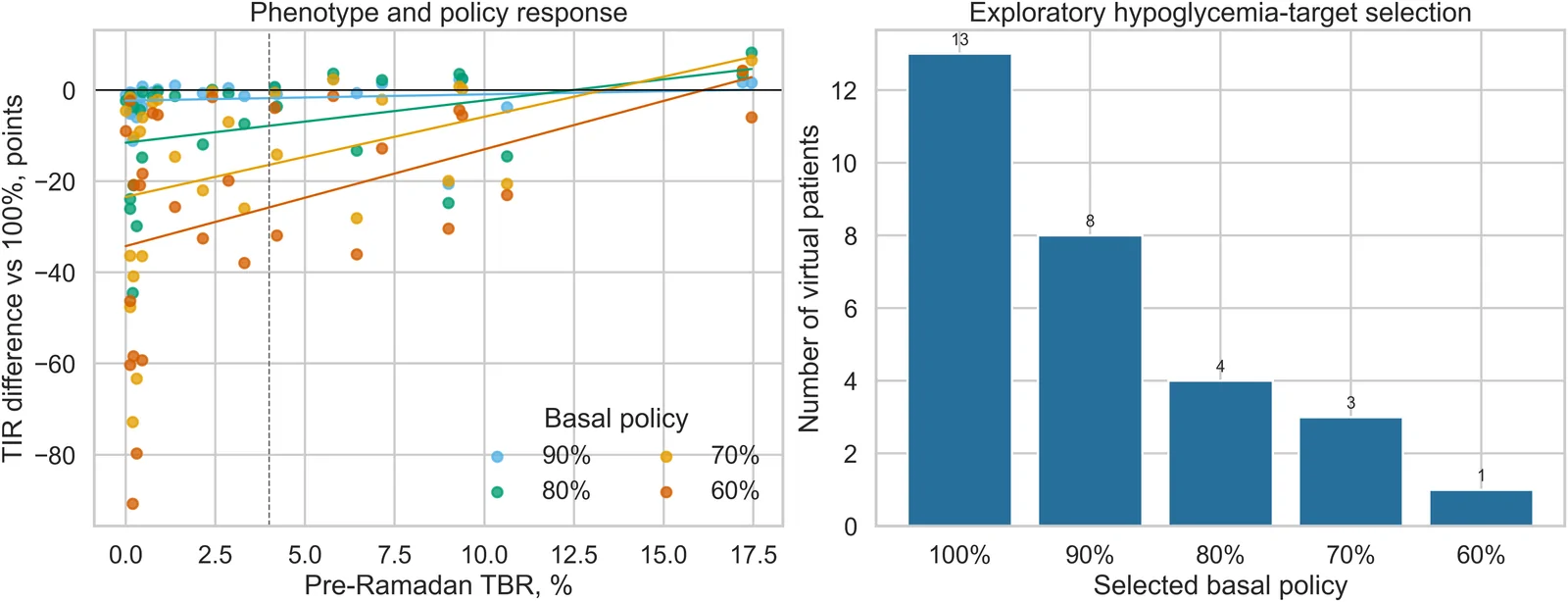
Exploratory phenotype-response and policy-selection analyses. Left: individual TIR effects relative to 100%; the dashed line marks the 4% pre-Ramadan TBR threshold. Right: policy maximizing TIR among policies meeting consensus TBR targets; TAR and rescue burden were not constrained.

The hypoglycemia-target-constrained exploratory rule selected 100% basal for 13 virtual patients, 90% for 8, 80% for 4, 70% for 3, and 60% for 1. Of the 18 virtual patients with pre-Ramadan TBR below 4%, 12 retained 100%, compared with 1 of 11 whose TBR was at least 4% (**Figure 2**). These in-sample selections are hypothesis-generating and do not constitute a validated decision algorithm.

### 3.4 Sensitivity analysis

The nine-day analysis including all 30 virtual patients reproduced the graded pattern. Mean TIR differences versus 100% were −1.9, −8.7, −17.7, and −27.8 points at 90%, 80%, 70%, and 60%, respectively. Corresponding TBR differences were −1.4, −2.3, −3.0, and −3.0 points. An exploratory age-group analysis (**Supplementary Figure 1**) showed the graded dose-response in children, adolescents, and adults alike, indicating the pattern does not depend on a single age group; group sizes were small, and these comparisons were underpowered.

### 3.5 Time-of-day decomposition localizes the trade-off

Splitting each Ramadan day into fasting-daytime (05:00–18:00), post-iftar (18:00–22:00), nocturnal (22:00–01:00), and suhoor (01:00–05:00) windows localized where basal reduction acts (**Supplementary Table 4**). Hypoglycemia was concentrated in the fasting daytime, where TBR fell from 6.6% at 100% basal to 0.7% at 60%, and the hourly hypoglycemia peak shifted from mid-fast (10:00; 13.2% of time below range at 100%) toward suhoor at deeper reductions. The greatest hyperglycemia burden was located in the post-iftar window and increased steeply as basal was reduced: post-iftar TAR rose from 40.2% at 100% to 76.1% at 60%, and post-iftar TAR *>* 250 from 13.7% to 43.8%. This pattern localizes the hyperglycemia burden to the post-iftar period and is consistent with a combined contribution from the meal–bolus model and the increasing basal deficit; because bolus timing and composition were not varied independently, the decomposition cannot separate the meal–bolus contribution from that of basal deficiency. What it does show is that basal reduction removes daytime fasting hypoglycemia while progressively worsening the already-dominant post-iftar hyperglycemia.

### 3.6 Severe hyperglycemia and terminal events by policy

Among the 29 complete-case virtual patients, severe hyperglycemia increased monotonically with reduction depth: mean TAR *>* 250 was 2.9%, 3.9%, 5.8%, 8.8%, and 14.8%, and TAR *>* 300 was 1.3%, 1.5%, 2.1%, 3.3%, and 5.9%, at 100%, 90%, 80%, 70%, and 60% basal, respectively (the corresponding all-30 safety-summary values in **Supplementary Table 5** are slightly higher, 3.6% to 15.1% for TAR *>* 250, because they additionally include child#008’s observed samples). Protocol-dependent terminal behavior was confined to a single virtual patient (child#008), which completed the 100% policy but terminated under every reduced policy; termination was earliest at the deepest reduction (day 9.8 at 60%), with similar times at 70–90% (days 24.8–25.8). This is consistent with greater vulnerability at the deepest reduction, while the similar termination times at 70–90% and the extreme trajectories also indicate simulator–protocol instability at glucose extremes rather than a clean dose-response in terminal timing. Policy-specific terminal-event rates were 0% at 100% and 3.3% (1/30) at each reduced policy. A worst-case analysis that treated the unobserved remainder of a terminated run as lost horizon increased the effective adverse burden monotonically with reduction depth (**Supplementary Table 5**), confirming that complete-case means modestly understate the risk of the deepest reductions.

### 3.7 The dose-response ordering is robust to reactive corrections

Because the primary analysis disabled routine reactive corrections to isolate the basal effect, we repeated all five policies with a realistic reactive-correction strategy (correction when CGM ≥250 mg/dL, dosing to a conservative 180 mg/dL target using each patient’s correction factor, a 4-hour refractory interval, and a patient-specific dose ceiling rather than a fixed cap). Enabling corrections raised TIR at the deepest policies (e.g., +5.9 points at 60%) and reduced severe hyperglycemia, but at the cost of a roughly doubled correction burden (from 27 to 43 corrections over 30 days at 60%), and it did not change the policy ordering: mean TIR remained monotonic at 81.5%, 80.5%, 75.8%, 68.3%, and 61.9% across 100% to 60% basal (**Supplementary Table 6**). The finding that 90% basal produced the smallest TIR loss among reduced policies while lowering TBR was therefore preserved when reactive corrections were enabled, and is not an artifact of disabling corrections.

### 3.8 Negative-control test of the pre-Ramadan marker

To test whether the pre-Ramadan TBR signal reflects a TBR-specific response or a generic “room-to-remove-hypoglycemia” floor effect, we repeated the omnibus effect-modification test with non-TBR pre-Ramadan markers as negative controls. The association was *not* specific to TBR. Pre-Ramadan TBR was strongly associated with response heterogeneity (mean |ρ|=0.50, omnibus P=0.007), but closely related baseline hypoglycemia/glycemia markers stratified the response to a comparable degree (LBGI, |ρ|=0.52, P=0.004; TIR, |ρ|=0.49, P=0.002), and even hyperglycemia markers used as shams showed weaker but non-null associations (TAR, |ρ|=0.33, P=0.025; HBGI, |ρ|=0.33, P=0.021) (**Supplementary Table 7**). This pattern is consistent with a broad baseline-glycemia or room-to-move component rather than a unique biological effect of TBR. We therefore interpret pre-Ramadan TBR as a clinically accessible candidate stratifier within a broader baseline-glycemia pattern—an exploratory, hypothesis-generating signal, not a validated predictive biomarker unique to TBR.

### 3.9 A safety-first selection rule shifts more patients toward reduction

The original descriptive selection rule fell back to the highest-TIR policy when consensus safety targets were unmet, which can favor an unsafe policy on TIR grounds. We therefore replaced this fallback with a lexicographic, safety-first rule that first restricts to policies meeting hypoglycemia safety thresholds (TBR *<* 70 ≤4%, TBR *<* 54 ≤1%, no terminal event) and then, when no policy qualifies, orders candidates by lowest TBR *<* 54, then lowest TBR, then lowest TAR, and only then highest TIR. Two evaluations followed. In-sample, relative to a TIR-first rule—which retained 100% basal in 17 of 29 patients—the safety-first rule retained 100% in only 12 of 29 and moved five additional patients to a protective reduction (agreement 76%), illustrating that a TIR-first fallback can favor a policy on TIR grounds that a safety-first rule would reject. We also re-derived this rule on training scenarios 1–2 and evaluated it on the held-out scenario 3; the held-out result—an explicit safety–TIR trade-off (hypoglycemia-target attainment 39% → 79%, TBR −2.9 points, at a TIR cost of −5.2 points)—is reported with its TIR-first comparator in the stochastic-scenario subsection below.

### 3.10 Sensor-noise robustness

Seed-averaged outcomes closely reproduced the primary results. Mean TIR was 81.9%, 80.0%, 73.9%, 65.5%, and 56.1% at 100%, 90%, 80%, 70%, and 60% basal, respectively, each within 0.2 points of **Table 1**. Paired differences from 100% were essentially unchanged (e.g., TIR differences of −1.9, −8.0, −16.4, and −25.8 points at 90%, 80%, 70%, and 60%). The phenotype association between pre-Ramadan TBR and the TIR effect remained significant at every reduced policy on seed-averaged data (𝜌 = 0.44 to 0.52, all *P <*0.05).

**Supplementary Table 3** summarizes descriptive between-patient and within-patient (across-seed) variance ratios. The within-patient ratio remained below 3% for TIR, TBR, TAR, and rescue corrections. For the rarer TBR below 54 mg/dL outcome, it increased to 11.9% at 70% basal and 22.4% at 60%, indicating greater relative sensor-seed sensitivity near zero exposure.

### 3.11 Meal-size associations depended on basal policy

**Figure 3:**
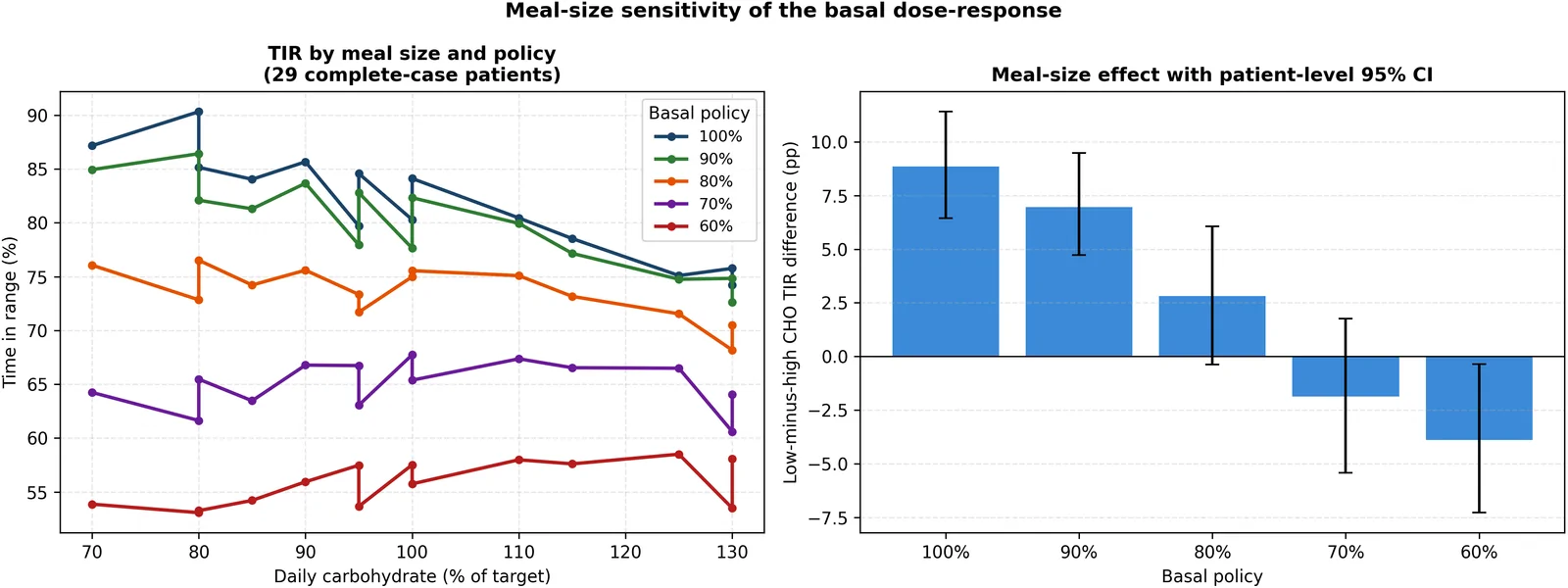
Meal-size sensitivity of the basal dose-response (29 complete-case virtual patients). Left: mean daily TIR by carbohydrate level for each policy. Right: within-patient low-minus-high carbohydrate TIR difference with patient-level 95% bootstrap confidence intervals. Low-carbohydrate days were associated with higher TIR at 100% and 90% basal (intervals excluding zero), were unresolved at 80% and 70%, and reversed at 60%, where basal deficiency dominates (patient-blocked ordered-trend *P <*0.001).

Among the 29 virtual patients completing all five 30-day policies, the within-patient difference in daily TIR between low-carbohydrate days (≤85% of target) and high-carbohydrate days (≥110%) depended strongly on the basal policy (**Figure 3**). Under mild reduction, low-carbohydrate days yielded higher TIR: the mean difference was 8.8 percentage points (95% CI 6.4 to 11.4) at 100% basal and 7.0 points (4.7 to 9.5) at 90%, both intervals excluding zero. At 80%, the difference was 2.8 points (95% CI −0.4 to 6.1) and at 70% it was −1.9 points (−5.4 to 1.8), neither resolved. At 60%, the difference reversed to −3.9 points (−7.3 to −0.4): under severe basal deficit, the larger meal bolus on high-carbohydrate days transiently drew some glucose into range. The patient-blocked ordered trend across policies was significant (Spearman 𝜌 = 0.57, *P <*0.001), indicating that meal size was associated with outcomes only when basal delivery was near-adequate. These results demonstrate a policy-dependent within-simulation association between carbohydrate exposure and TIR; they do not establish the clinical effectiveness of meal-size counseling.

### 3.12 Stochastic meal/timing scenarios reproduced the dose-response

All six policies were completed together in 82 of 87 patient-scenario blocks, with 28 patients contributing at least one paired-complete block; the remaining 26 terminal runs clustered in three models (adolescent#007, adolescent#008, child#003) and were reported separately rather than truncated.

**Figure 4:**
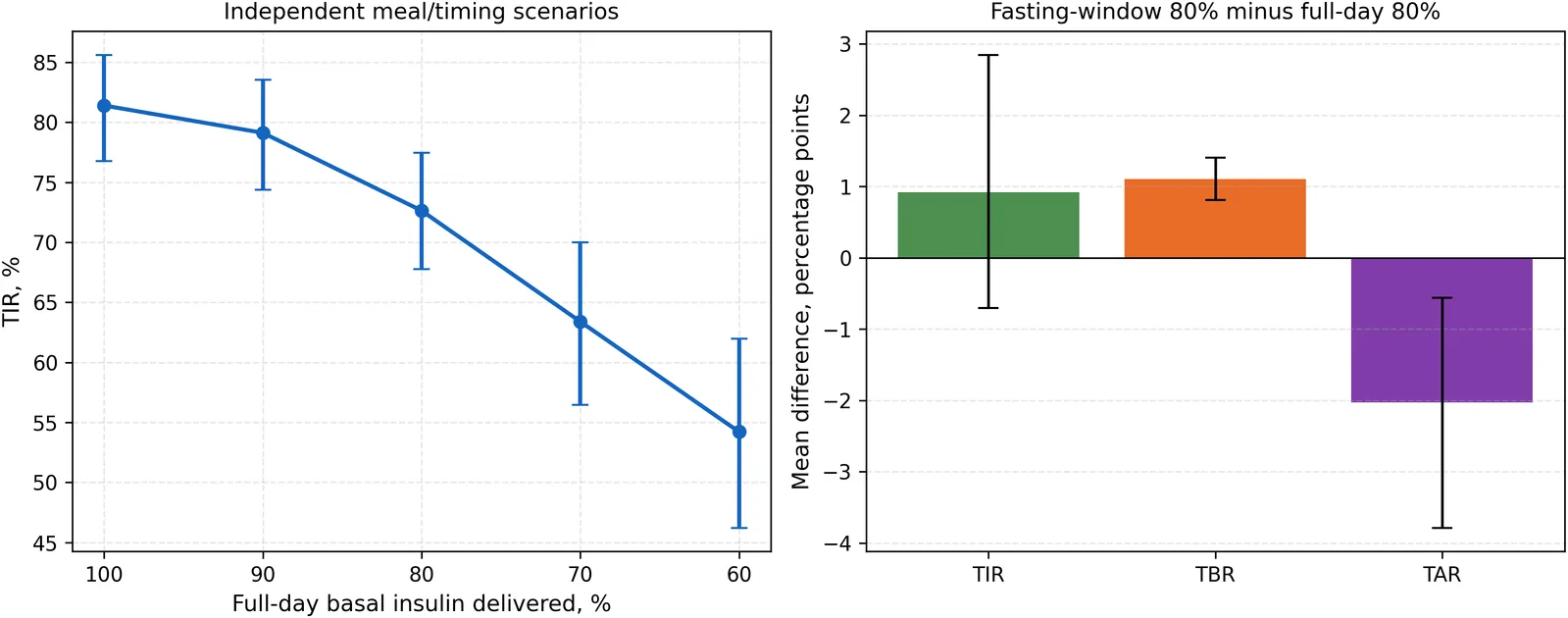
Meal/timing stochastic extension. Bars and points show paired means; whiskers show patient-level bootstrap 95% confidence intervals. Left: mean TIR for full-day policies. Right: paired mean differences for fasting-window 80% minus full-day 80%; positive TBR denotes more hypoglycemia and negative TAR denotes less hyperglycemia.

The full-day dose-response was reproduced after averaging each patient’s paired-complete stochastic scenarios (**Table 3**). Compared with full-day 80%, fasting-window 80% changed TIR by 0.9 percentage points (95% CI −0.7 to 2.8; adjusted P=0.348), TBR by 1.1 points (0.8 to 1.4; adjusted P<0.001), TAR by −2.0 points ( −3.8 to −0.6; adjusted P=0.011), and rescue corrections by −0.5 per 30 days ( −1.2 to 0.1; adjusted P=0.143). Thus, limiting the reduction to fasting hours shifted exposure from hyperglycemia toward hypoglycemia without a statistically significant TIR gain (**Figure 4**).

**Table 3:** Outcomes under independent meal/timing scenarios.

Policy	TIR, %	TBR, %	TBR *<* 54, %	TAR, %	Rescue, n
Full-day 100%	81.4	5.2	1.5	13.4	6.5
Full-day 90%	79.1	3.6	1.0	17.3	8.9
Full-day 80%	72.6	2.4	0.5	25.0	13.2
Full-day 70%	63.4	1.6	0.3	35.0	18.8
Full-day 60%	54.2	1.0	0.1	44.7	28.6
Fasting-window 80%	73.6	3.5	0.9	23.0	12.8

Values are patient means after averaging all paired-complete scenarios available for each of 28 virtual patients. Terminal runs were excluded from continuous summaries and are reported in the text.

The scenarios 1–2 selection rule chose 100% basal for 18 patients, 90% for 5, 80% for 4, and 60% for 1, with 17 of 28 patients having at least one policy that met all three consensus CGM targets. In held-out scenario 3, the locked rule versus 100% changed TIR by 0.6 points (95% CI 0.1 to 1.1; adjusted P=0.078), TBR by −1.6 points (−2.8 to −0.7; adjusted P=0.009), TBR below 54 mg/dL by −0.7 points (−1.5 to −0.2; adjusted P=0.009), and TAR by 1.1 points (0.4 to 1.8; adjusted P=0.009); rescue corrections increased by 1.5 per 30 days (0.0 to 4.0; adjusted P=0.125). Joint attainment of all three CGM targets rose from 35.7% under uniform 100% basal to 46.4% under this comparator selection. This TIR-first comparator thus reduced hypoglycemia exposure in the held-out scenario without a statistically detected reduction in TIR (this was not an equivalence or non-inferiority evaluation).

Because the TIR-first comparator is permitted to fall back on the highest-TIR policy—the behavior the reviewer flagged—we adopted a safety-first rule as our primary held-out analysis and re-derived it on scenarios 1–2. Applied to held-out scenario 3, the safety-first rule sent 11 of 28 patients to the lexicographic safety ordering; relative to uniform 100% basal it reduced TBR by 2.9 points (95% CI −4.4 to −1.7) and TBR below 54 mg/dL by 1.1 points (−1.9 to −0.5), raising the proportion of patients meeting the hypoglycemia targets from 39% to 79%, but at a TIR cost of −5.2 points (−9.1 to −1.7) and a TAR increase of 8.1 points. This makes the trade-off explicit: prioritizing fasting hypoglycemia safety necessarily sacrifices some TIR, and the balance is a clinical rather than a purely statistical choice. Both rules were transported within the same virtual patients (not across new physiology or clinical data); we foreground the safety-first rule as the more defensible choice for a fasting context.

## 4 Discussion

This five-policy experiment identifies a clear dose-response relation. Each incremental basal reduction decreased hypoglycemia exposure, but reductions of 20% or more caused progressively larger TIR loss, higher TAR, and greater rescue burden. The 90% policy occupied a distinct region of the trade-off, reducing TBR while preserving group-level TIR far better than aggressive policies. This does not establish clinical superiority, but it supports prospective evaluation of mild basal reductions centered around the 90% policy, rather than the larger uniform reductions of 20–40% that are commonly recommended as defaults.

The simulated pattern is consistent with real-world CGM observations during Ramadan. Observational cohorts of adults with type 1 diabetes have reported that the largest glucose excursions occur after iftar and suhoor and that routine insulin adjustments tend to be insufficient around these meals, with glycemic deterioration concentrated in those with poorer baseline control (5, 6). Our results offer a mechanistic interpretation of these clinical observations: when basal delivery is held near-adequate, the dominant residual driver of excursions is the carbohydrate-dense iftar, so that the marginal value of basal reduction is small and chiefly affects fasting-hour hypoglycemia; once basal is reduced substantially, the resulting daytime insulin deficit propagates into the post-meal period and amplifies hyperglycemia. This is the same direction of effect reported when structured education and advanced monitoring, rather than basal reduction alone, were required to fast safely (14), and when alternate pump and bolus settings outperformed basal adjustment in a randomized pilot (13). The convergence between our simulated dose-response and these independent clinical reports supports the plausibility of the mechanism, while underscoring that basal reduction is one lever among several.

The phenotype result was consistent across all reduced policies. Virtual patients with little pre-Ramadan hypoglycemia had limited TBR available to reduce and incurred a disproportionate TIR penalty. Those with elevated pre-Ramadan TBR tolerated moderate reductions more favorably. The hypoglycemia-target-constrained selection analysis consequently retained 100% for most low-TBR virtual patients and selected reduced policies more often in the high-TBR group. This association should be interpreted cautiously: it may partly reflect differential room for hypoglycemia reduction — patients with higher baseline TBR simply have more TBR to remove — rather than a distinct biological responsiveness. It should therefore be regarded as a candidate stratification signal that is hypothesis-generating, not a validated predictive biomarker, and requires confirmation in independently identified cohorts or clinical cohorts.

The independent meal/timing scenarios reproduced the dose-response and the phenotype association despite daily variation in carbohydrate amount, allocation, and timing, and allowed two locked selection strategies to be contrasted. Fasting-window 80% reduced TAR relative to full-day 80% but yielded no resolved TIR advantage. The TIR-first comparator reduced hypoglycemia without a statistically detected TIR reduction (+0.6 points; adjusted *P* = 0.078), but retained the highest-TIR fallback criticized during review. The revised safety-first rule produced a larger reduction in TBR and substantially improved hypoglycemia-target attainment (39% to 79%), but incurred a 5.2-point TIR cost. Together, these results provide internal scenario-transport evidence while demonstrating that the balance between fasting safety and TIR preservation depends on the priority embedded in the selection rule. Neither analysis constitutes validation in new physiology or clinical data.

Clinical interpretation requires caution, as Ramadan management is multifactorial: alternate pump settings and advanced bolus features improved TIR over basal adjustment alone (13), CGM-guided temporary basal reductions have been used safely (17), and hybrid closed-loop therapy benefits higher-risk fasters (7, 11). The rising rescue burden at lower policies indicates that basal reduction cannot be separated from post-iftar bolus strategy and fast-termination rules; indeed, crossover trials of the traditional iftar meal show that maintaining postprandial euglycemia requires specific, often dual-wave, bolus patterns rather than basal adjustment (29), and adjunctive agents that blunt the post-iftar surge can further raise TIR (30). Future policy spaces should therefore combine time-localized basal changes with adaptive post-iftar correction rather than simply extending the basal dose range.

The meal-size analysis revealed a significant within-simulation ordered trend (*P <*0.001): lower carbohydrate intake was associated with higher TIR under mild reduction (7–9 points at 90%–100% basal) but not under aggressive reduction. Mechanistically, when basal delivery is near-adequate the post-iftar carbohydrate load dominates excursions, whereas under large reductions the insulin deficit dominates and meal moderation no longer helps. Because daily carbohydrate followed a prespecified sequence rather than independent randomization, this is a within-simulation association, not causal evidence; it suggests meal-size moderation as a candidate co-intervention for prospective testing under mild reduction, not a standalone recommendation.

The four terminal events in one virtual patient are also informative: the same model completed 100% basal despite severe excursions yet failed under every reduced policy without a monotonic time to failure, highlighting both individual vulnerability and simulator instability at extreme glucose. Because excluding it could make reduced policies appear safer, we reported the events separately and confirmed the pattern in an all-patient sensitivity analysis.

### 4.1 Strengths and limitations

The principal strengths of this study follow from its paired, multi-policy design. Comparing five basal policies within the same virtual physiology, under an identical meal stream and sensor-noise seed, removes between-patient and meal-stream confounding that limits single-policy and cross-patient analyses, and allows the full dose-response shape to be traced rather than a single contrast. The design is further supported by 30-day horizons rather than single days, patient-level bootstrap inference, three independently seeded meal/timing scenarios, a scenario-held-out policy evaluation, a prespecified meal-size sensitivity analysis with a patient-blocked ordered-trend test, and a formal patient-blocked test of phenotype effect modification. All simulation and analysis code, derived datasets, and figures are openly archived, so that every reported number can be regenerated from fixed seeds.

Important limitations remain and bound the interpretation. The sensor-noise robustness check showed within-patient variance ratios below 3% for TIR, TBR, TAR, and rescue corrections, but higher ratios for TBR below 54 mg/dL under the most aggressive reductions (**Supplementary Table 3**), so that rare severe-hypoglycemia exposure was more sensitive to the specific noise draw than the headline outcomes. Only three meal/timing scenarios were sampled, all from prespecified distributions, and terminal events reduced some scenario blocks; broader distributions of food composition, fasting duration, physical activity, illness, and behavioral adherence were not modeled. The bolus model also does not represent pre-bolus timing, dual-wave or square-wave bolus patterns, or the delayed glycemic effect of the fat- and protein-rich iftar—features shown to be important for post-iftar euglycemia (29)—so the post-iftar hyperglycemia estimates and the policy-dependent meal-size association should be read as within-simulation associations rather than causal evidence for meal counseling. Most fundamentally, the 29 complete-case virtual patients are a fixed simulator library rather than a random sample of a clinical population; consequently, the bootstrap intervals and permutation *P* values quantify uncertainty across heterogeneity within the simulator cohort and must not be read as direct statistical inference to people with type 1 diabetes. Findings derive from a single physiological model family (UVA/Padova), and the policy-selection rule was held out by scenario but not by virtual patient, simulator family, or clinical cohort. The meal-size sequence was prespecified rather than independently randomized, so meal size may be partly confounded with study day and timing; the resulting interaction is a within-simulation association, not causal evidence for meal counseling. No clinical CGM dataset was available for external validation. Finally, the simulation omits dehydration, sleep disruption, variable activity, intercurrent illness, and imperfect adherence; carbohydrate was assumed known perfectly, and only a single fasting-window dose was evaluated. Routine reactive corrections were disabled in the primary analysis to isolate the basal lever, but a realistic reactive-correction sensitivity analysis confirmed that the policy ordering was preserved. Evening physical activity associated with Taraweeh prayers, which can influence post-iftar and nocturnal glycemia, was not represented. Each of these simplifications would need to be relaxed before any clinical inference could be drawn.

Two directional caveats deserve particular emphasis. First, the UVA/Padova model was developed and accredited for closed-loop and sensor evaluation and has not been validated specifically for the physiology of prolonged, repeated Ramadan fasting. It does not represent progressive glycogen depletion, the blunting of counterregulatory responses over successive fasting days, dehydration-related changes in insulin absorption, or circadian shifts in insulin sensitivity—all of which would tend to *increase* real-world fasting hypoglycemia beyond that simulated here. If so, the model would *underestimate* the protective value of basal reduction, and the apparent advantage of the mild (90%) policy must not be read as evidence that a 10% reduction is clinically sufficient; the true optimum in fasting patients at elevated hypoglycemia risk may lie at a deeper reduction than the simulation suggests. Second, and relatedly, all findings pertain to continuous *pump* basal-rate modulation and must not be extrapolated to long-acting basal insulin in MDI regimens, where the fasting-window concept is not physically realizable and dose changes act with a multi-day lag.

### 4.2 Clinical translation and future directions

These findings are hypothesis-generating and define a concrete translational pathway rather than a dosing rule. The most actionable signal is that, among reduced-basal policies, a mild 10% reduction produced the smallest TIR loss while still reducing hypoglycemia, and that pre-Ramadan TBR—a quantity already available from routine CGM before Ramadan—stratified the response. A prospective study could therefore stratify participants by pre-Ramadan TBR and compare a small set of basal policies centered on the mild-reduction region, using the same CGM consensus targets as endpoints. Because the rescue burden rose steeply at lower policies, such a study should pair any basal change with a defined post-iftar bolus and correction strategy rather than varying basal in isolation. The meal-size interaction suggests that dietary counseling on iftar portion size may add the most value precisely in the mild-reduction group, a question that a factorial clinical design could address directly. Methodologically, the next in silico step is to lock the CGM-target selection rule and evaluate it in an independently identified virtual cohort or a second simulator family, and ultimately to anchor it to real pre-Ramadan CGM through patient-specific model identification, held-out prediction testing, and prospective safety evaluation.

### 4.3 Research implications

A confirmatory in silico study should cross several fasting-window doses with adaptive post-iftar correction across a broader scenario distribution, and use hierarchical models to separate between-patient heterogeneity from within-patient scenario uncertainty. The CGM-target rule should be locked before evaluation in a held-out simulator family or independently identified cohort, then anchored to clinical CGM; clinical translation would require patient-specific model identification, held-out prediction testing, and prospective safety evaluation before any change to practice.

## 5 Conclusions

Pump basal-rate reduction during simulated Ramadan fasting produced a graded hypoglycemia–hyperglycemia trade-off rather than a single optimal dose. Among reduced-basal policies, a mild 10% reduction produced the smallest TIR loss while still lowering hypoglycemia, and the price of each further reduction in TIR, severe hyperglycemia, and treatment burden accelerated. Pre-Ramadan TBR, a routinely available CGM quantity, stratified the TIR response in a formal patient-blocked test—though a negative-control analysis showed this signal was not unique to TBR but part of a broader baseline-glycemia pattern. In a held-out scenario, a revised safety-first policy-selection rule improved hypoglycemia-target attainment and reduced TBR but at an explicit TIR cost, demonstrating that prioritizing fasting safety involves a glycemic trade-off rather than a cost-free gain. Meal size and basal policy interacted, with carbohydrate moderation associated with higher TIR only when basal delivery remained near-adequate. Taken together, these results suggest that Ramadan basal adjustment is best viewed not as a uniform default but as a phenotype-dependent choice within a mild-reduction region, combined with attention to iftar meal size and post-iftar dosing. Because these analyses use a fixed simulator library rather than a random clinical sample, the reported intervals and *P* values characterize heterogeneity within the simulated cohort and do not support population-level inference. The study does not propose a clinical dosing rule; rather, it identifies less favorable simulated policy regions, a mild-reduction region with the smallest simulated TIR loss, and a candidate CGM-based stratifier—all hypothesis-generating outputs that require prospective clinical evaluation before any change to practice.

## Data Availability

The datasets presented in this study can be found in online repositories. The names of the repository/repositories and accession number(s) can be found below: Zenodo (permanent archive), version v5.0.0, DOI: https://doi.org/10.5281/zenodo.21402118; GitHub (source repository): https://github.com/ameen-banjar/ramadan-basal-policy-simulation.
