# Supplementary material for: Pre-Ramadan time-below-range stratifies the simulated pump basal-rate dose–response during Ramadan fasting: a five-policy paired virtual-patient study
Source: Front Endocrinol (Lausanne). 2026 Sep 4;17:1922149. doi: 10.3389/fendo.2026.1922149 (PMC13585517; doi:10.3389/fendo.2026.1922149)
Supplement: Supplementary file 1 [file DataSheet1.pdf]

Supplementary Material

Pre-Ramadan Time-Below-Range Stratifies the Simulated Pump Basal-Rate Dose–Response During Ramadan Fasting:

A Five-Policy Paired Virtual-Patient Study

Ameen Banjar

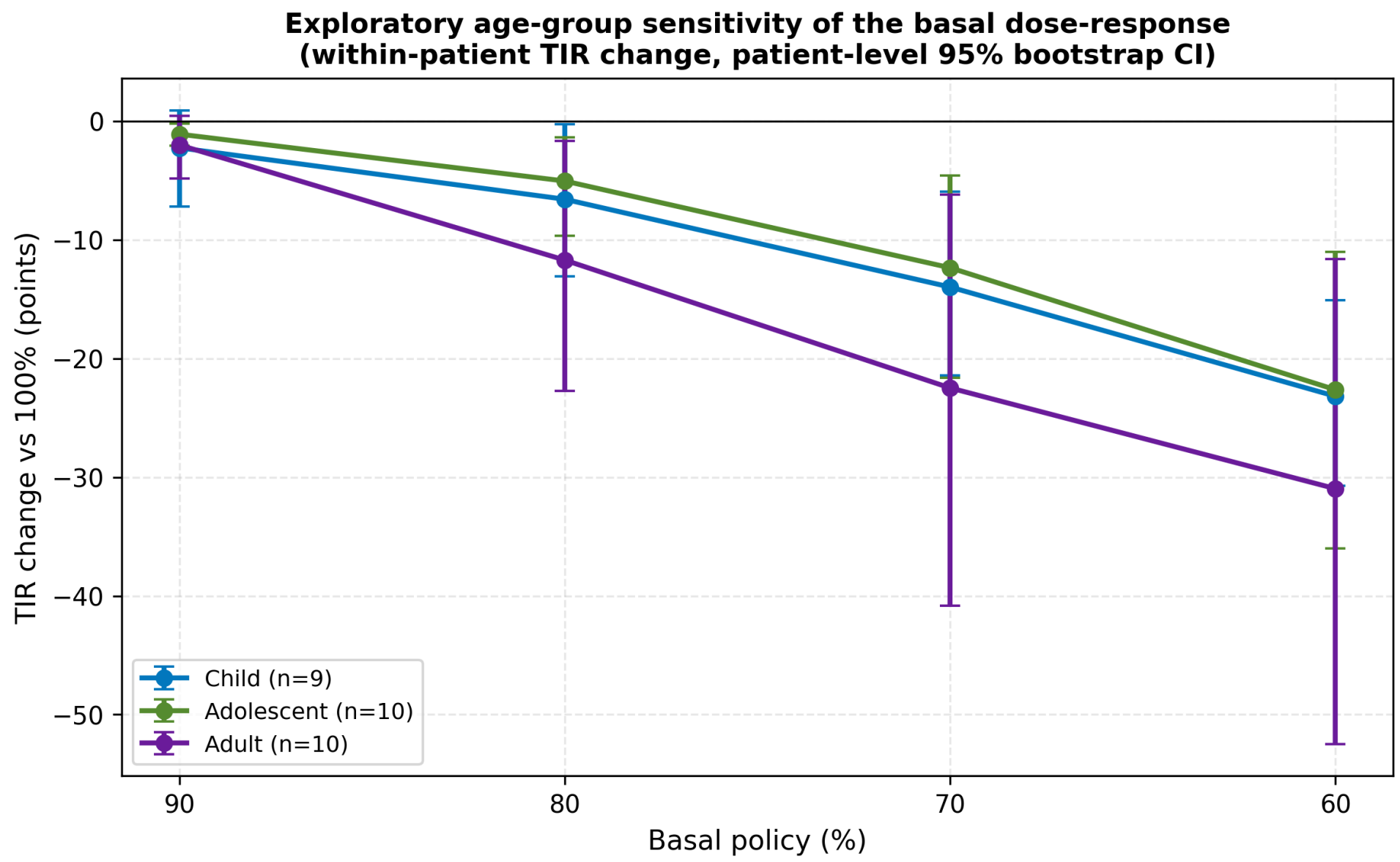

Figure S1: Exploratory age-group sensitivity of the basal dose-response. Within-patient TIR change from the 100% policy by age group (9 children, 10 adolescents, 10 adults), with patient-level 95% bootstrap confidence intervals. The graded dose-response is present in all three groups, indicating that the overall pattern does not depend on a single age group. Group sizes are small and these analyses are exploratory and underpowered for formal age comparisons.

Table S1: Virtual cohort characteristics

Age group	*n*	Age, mean (range)	Basal, U/h	ICR, g/U	ISF, mg/dL/U
Child	10	9.4 (7–12)	0.41	18.7	34.5
Adolescent	10	16.5 (14–19)	0.76	12.0	19.8
Adult	10	51.6 (26–68)	1.25	10.3	16.2

ICR, insulin-to-carbohydrate ratio; ISF, insulin sensitivity factor.

Table S2: Safety profile of `child#008` across policies

Policy	Terminal	Days observed	Min BG	Max BG	Safety corrections
100%	No	30	15.1	591.2	51
90%	Yes	26	19.1	599.3	45
80%	Yes	25	29.9	602.0	43
70%	Yes	25	34.4	597.7	43
60%	Yes	10	59.8	600.8	19

BG in mg/dL. A terminal run reflects the simulator's physiological stopping condition (BG >600 or <10 mg/dL). This virtual patient completed 100% basal but terminated under every reduced policy, with earlier termination at the deepest reduction (day 10 at 60%). The behavior reflects both a dose-dependent vulnerability and simulator–protocol incompatibility at extreme glucose states, and should not be read as a clinically calibrated event incidence (see also Supplementary Table S5).

Table S3: Sensor-noise robustness: descriptive within-patient across-seed variance ratio

Outcome	100%	90%	80%	70%	60%
TIR, %	0.4	0.6	0.3	0.1	0.1
TBR < 70, %	0.6	0.8	1.0	1.5	2.9
TBR < 54, %	1.7	1.7	2.9	11.9	22.4
TAR > 180, %	0.4	0.6	0.3	0.1	0.1
Rescue corrections, n	1.0	1.1	0.7	0.6	0.2

Values are a descriptive ratio: mean within-patient (across-seed) variance divided by that quantity plus the variance of patient seed averages, expressed as a percentage. They are not mixed-model variance components. Results are based on three independent CGM sensor-noise seeds per patient-policy pair among the 29 complete-case virtual patients.

Table S4: Time-of-day glycemic decomposition by basal policy (mean over the 29 complete-case virtual patients). Windows: fasting daytime (05:00–18:00), post-iftar (18:00–22:00), nocturnal (22:00–01:00), suhoor (01:00–05:00).

Policy	Window	TIR, %	TBR, %	TAR, %	TAR>250, %
100%	Fasting daytime	87.6	6.6	5.8	1.0
	Post-iftar 0–4 h	59.7	0.2	40.2	13.7
	Nocturnal	79.2	4.0	16.9	2.3
	Suhoor	81.5	4.9	13.6	2.5
90%	Fasting daytime	87.9	4.2	7.9	1.5
	Post-iftar 0–4 h	50.8	0.2	49.0	17.8
	Nocturnal	75.9	3.3	20.9	2.5
	Suhoor	81.2	3.5	15.3	2.5
80%	Fasting daytime	82.1	2.4	15.5	2.7
	Post-iftar 0–4 h	40.3	0.2	59.5	24.0
	Nocturnal	71.9	2.5	25.6	3.1
	Suhoor	78.6	2.7	18.7	3.1
70%	Fasting daytime	72.4	1.3	26.3	4.7
	Post-iftar 0–4 h	30.8	0.1	69.1	33.6
	Nocturnal	66.4	1.9	31.8	4.4
	Suhoor	73.5	1.8	24.7	3.6
60%	Fasting daytime	61.2	0.7	38.0	11.0
	Post-iftar 0–4 h	23.8	0.1	76.1	43.8
	Nocturnal	61.5	1.6	36.9	7.0
	Suhoor	66.9	1.4	31.7	5.7

Hypoglycemia is concentrated in the fasting daytime and falls with reduction; the greatest hyperglycemia burden is located in the post-iftar window and increases steeply as basal is reduced. Because bolus timing and composition were not varied independently, this decomposition localizes the burden but does not isolate meal–bolus from basal-deficit contributions.

Table S5: Severe hyperglycemia, policy-specific terminal events, and worst-case horizon loss by basal policy.

Policy	TAR>180, %	TAR>250, %	TAR>300, %	Terminal events, n (%)	Lost horizon, %[a]
100%	14.2	3.6	1.3	0/30 (0.0)	0.00
90%	17.6	4.5	1.5	1/30 (3.3)	0.47
80%	24.6	6.4	2.1	1/30 (3.3)	0.58
70%	33.9	9.3	3.3	1/30 (3.3)	0.58
60%	43.2	15.1	5.9	1/30 (3.3)	2.24

TAR values in this table are computed over all 30 virtual patients (each patient's observed period), so they differ slightly from the complete-case ($n = 29$) values in the main text (e.g., TAR>250 2.9%→14.8% for $n = 29$ here 3.6%→15.1% for $n = 30$); the difference is the inclusion of child#008's observed samples. Terminal behavior was confined to child#008, which completed 100% basal but terminated under every reduced policy, with the earliest termination at the deepest reduction (day 9.8 at 60%; days 24.8–25.8 at 70–90%).

[a] Worst-case sensitivity: mean fraction of the 30-day horizon lost to termination, treated as adverse; it increases monotonically with reduction depth.

Table S6: Reactive-correction sensitivity analysis: five policies re-run with a realistic reactive-correction strategy (correction at CGM ≥ 250 mg/dL to a 180 mg/dL target using each patient's correction factor, a 4-hour refractory interval, and a patient-specific dose ceiling). Means over the 29 complete-case virtual patients.

Policy	TIR, %	TBR, %	TBR<54, %	TAR, %	TAR>250, %	Corrections, n
100%	81.5	5.5	1.4	13.1	2.8	18.0
90%	80.5	3.8	0.9	15.6	3.4	21.2
80%	75.8	2.6	0.5	21.6	4.2	25.6
70%	68.3	1.8	0.3	29.9	6.0	33.9
60%	61.9	1.2	0.2	37.0	7.8	43.1

Enabling corrections raised TIR at the deepest policies and reduced severe hyperglycemia, but roughly doubled the correction burden, and did not change the monotonic policy ordering (compare the primary results in the main text).

Table S7: Negative-control (sham) test of pre-Ramadan effect modification. Omnibus patient-blocked permutation test of whether each pre-Ramadan marker modifies the TIR dose-response across the four reduced policies.

Pre-Ramadan marker	Role	Mean $\lvert\rho\rvert$	Omnibus P
TBR (<70 mg/dL)	Primary hypothesis	0.50	0.007
TAR (>180 mg/dL)	Sham / negative control	0.33	0.025
HBGI	Sham / negative control	0.33	0.021
LBGI	Hypoglycemia-related	0.52	0.004
TIR	Glycemia-related	0.49	0.002

Pre-Ramadan TBR (and closely related hypoglycemia markers, LBGI/TIR) show the strongest, most consistent association. A hyperglycemia sham marker (TAR, HBGI) shows a weaker but non-null association, indicating a partial floor/room-to-move component. Pre-Ramadan TBR is therefore an exploratory candidate stratification signal, not a validated predictive biomarker.
